# Effectiveness of a remedial educational program on enhancing spelling skills in primary school students with learning disabilities

**DOI:** 10.3389/fpsyg.2026.1714351

**Published:** 2026-02-04

**Authors:** Ayman Abdullah Hazza Alamri

**Affiliations:** Education College, Department of Special Education, Prince Sattam Bin Abdulaziz University, Al-Kharj, Saudi Arabia

**Keywords:** learning disabilities, remedial educational program, spelling, intervention, language

## Abstract

**Introduction:**

Learning disabilities significantly affect students’ academic performance, particularly in spelling proficiency among primary school learners. This study aimed to develop and validate the Learning Disabilities Spelling Scale (LDSS) and to evaluate the effectiveness of a remedial educational intervention designed to enhance spelling skills in students with learning disabilities.

**Methods:**

A quasi-experimental design with experimental and control groups was employed. 24 primary school students diagnosed with learning disabilities participated in the study and were assigned to an experimental group and a control group. The LDSS was administered at three time points to examine both immediate and sustained effects of the intervention on spelling performance.

**Results:**

Findings revealed statistically significant differences in post-test in favor of the experimental group compared with the control group, indicating the effectiveness of the remedial educational program. In addition, there were no statistically significant differences between the experimental group’s post-test and follow-up scores, supporting the sustained positive outcomes associated with the intervention.

**Discussion:**

Findings support the use of the structured remedial educational programs to improve and sustain spelling performance. These results underscore the importance of targeted assessment and intervention in addressing literacy-related learning disabilities in primary education and highlight implications for practice and future research on remediation and progress monitoring.

## Introduction

Learning disabilities (LD) are neurological disorders affecting an individual’s ability to acquire, process, store, and use information effectively (Fletcher et al., 2018). These disorders may impact various cognitive processes involved in learning, such as reading, writing, spelling, reasoning, and mathematical skills (Grigorenko et al., 2020). It is essential to note that learning disabilities do not indicate a lack of intelligence or motivation. Instead, individuals with learning disabilities often have average to above-average intelligence but encounter difficulties in specific areas of learning (Sleeter, 2018).

It is important to distinguish LD from specific learning difficulties (SPLD), a term commonly used in educational and clinical contexts to refer to particular, circumscribed difficulties within the broader LD category. SPLD include conditions such as dyslexia (reading and language processing difficulties), dysgraphia (writing and transcription difficulties), and dyscalculia (mathematical reasoning difficulties) (Prior, 2022). While LD represents an overarching diagnostic framework, SPLD refers to the specific manifestation of learning-related impairments within that framework (American Psychiatric Association, 2013; Fletcher et al., 2018). The causes of LD and SPLD are multifactorial, involving genetic, neurological, and environmental influences, and these conditions typically persist across the lifespan. However, early identification and targeted educational interventions can significantly reduce their academic and functional impact (Grigorenko et al., 2020). Consequently, teachers and special education professionals play a critical role in recognizing learning disabilities and implementing individualized instructional strategies and accommodations that support students’ academic success.

### Spelling skills among students with learning disabilities

Spelling is essential in both writing and reading. Misspelled words can make writing more difficult to read, causing readers to depreciate the quality of a writer’s message (Graham et al., 2011), so mastering spelling is crucial for students as it positively impacts reading and expressive writing outcomes (Kohnen et al., 2010). It was discovered that teachers assigned lower marks to papers with misspelled words than to those without, based on the quality of the presented concepts. When comparing a document with 3–13% misspelled words to the same version without any spelling errors, the quality of the content was rated lower by 0.38 standard deviation points (Graham and Santangelo, 2014).

Correct spelling is a fundamental linguistic skill and a key component of written language development that supports clear and accurate communication (Ciullo et al., 2025). Spelling is typically defined as the ability to construct words using letters in accordance with the orthographic conventions of a given language and relies on the coordinated use of multiple linguistic systems (Treiman and Kessler, 2014; Adoniou, 2014). To become proficient spellers, students must draw on phonological awareness, morphological knowledge, visual memory for letter patterns, and an understanding of semantic and, in some cases, etymological relationships among words (Adoniou, 2014; Saiegh-Haddad and Taha, 2017). Consequently, many of the linguistic processes that underpin successful reading—such as decoding and morphological processing—are also essential for accurate spelling, highlighting the close developmental linkage between reading and spelling skills (Graham and Santangelo, 2014; Kim et al., 2015).

Students who have trouble spelling words are likely to have trouble reading as well, and vice versa. The correlation between reading and spelling difficulties in students with LD and learning challenges is evident, as is the co-morbidity of these issues (Lazarus, 2016). Additionally, during the writing process, spelling proficiency is directly related to written composition fluency. In addition to writing quality papers, students with high spelling skills can read written materials with ease. Similarly, those who struggle with spelling could also find it challenging to write well and fluently (Lazarus and Audu, 2023).

Spelling is one of the most challenging areas students with LD face, and improving spelling outcomes for these students is of high importance. Those with LD struggle to spell vocabulary appropriate for their grade level, straining educational systems and compromising the learning process due to the prevalence of these issues (Fletcher et al., 2018). Visual perception disorders in LD result in difficulties with word and letter recognition, affecting reading and spelling due to impaired visual processing (Saqr, 2017). Challenges in alphabet memorization may stem from irregular alphabet patterns, complicating the learning process for some students (Al-Zayat, 1998). Research indicates that while typical students spell age-appropriate vocabulary accurately, students with LD in reading and writing continue to experience spelling difficulties, contributing to higher rates of academic failure among children and adolescents (Gordon et al., 1993; McNaughton et al., 1994; Simonsen and Dixon, 2004).

The ability to spell relies on understanding the relationship between sounds and letters, aiding the child in the writing process, whereas a lack of awareness of this connection leads to spelling difficulties and inaccuracies in writing (Vaughn et al., 2011). Spelling difficulties stem from poor visual memory of word forms and struggles in phonetic recall, as accurately spelling a word necessitates remembering its correct form (Mohammed, 2007). Consequently, students with LD often exhibit weaker spelling abilities compared to younger, typically developing peers (Friend and Olson, 2008). They face heightened challenges in spelling due to two primary factors: (a) difficulties in recognizing word sounds (Wendling and Mather, 2009); and (b) struggle in applying their spelling skills across various contexts (Wanzek et al., 2006).

### Remedial educational programs for spelling skills

Remedial educational programs can be described as structured, targeted forms of extra instruction that are offered to students who are not making adequate progress through typical classroom teaching, with the goal of reducing specific academic deficits and improving participation in the regular curriculum (Buckingham, 2020; Speld QLD, 2025). They emerge as tailored interventions designed to address the specific academic challenges encountered by students, particularly those with LD (Ferraz et al., 2018). In the context of spelling skills for primary school students facing LD, these programs typically include targeted instructional strategies, adaptive learning resources, and personalized support structures (Fragkouli et al., 2022). These programs usually concentrate on core component skills-such as phoneme–grapheme links, word recognition, and common orthographic patterns-and rely on clearly organized, explicit teaching routines delivered in small-group or individual formats so that practice, support, and feedback can be closely tailored to learners’ needs (Castles et al., 2018; McArthur et al., 2018). Recent accounts also emphasize that effective remedial interventions often integrate multisensory techniques and a sequence of tasks that move from highly controlled to more complex reading and writing activities, enabling students to apply and consolidate their developing skills over time (Saleem and Arshad, 2025; Speld QLD, 2025).

LD encompass a varied spectrum of neurological conditions that impact information acquisition, processing, and retention (Williams et al., 2017). These conditions, characterized by challenges in specific cognitive processes, do not reflect a lack of intelligence but present difficulties in particular areas of learning. Spelling skills, a fundamental component of literacy, can be notably affected in students with LD (Willcutt et al., 2019). Improving spelling outcomes for these students is of high importance, as proficiency in spelling is crucial for effective communication and the development of essential reading and writing skills (Kelly et al., 2017). Consequently, difficulties in mastering spelling can influence academic performance across diverse subjects. The primary objective of this study is to equip students with the tools and strategies necessary to overcome spelling difficulties and achieve academic success.

Recent research has continued to demonstrate that well-designed spelling interventions can produce meaningful gains for students with, or at risk for, LD. Meta-analytic and systematic review evidence indicates that explicit, systematically sequenced spelling instruction, particularly when it targets phoneme–grapheme correspondences and orthographic patterns, leads to significant improvements in spelling accuracy and can also support growth in word reading (Chandler et al., 2025; Galuschka et al., 2020; Murnan, 2023). Studies focusing on children with LD suggest that combining phonics teaching with structured spelling practice enhances both letter–sound knowledge and word-level spelling performance, especially when instruction is delivered explicitly and cumulatively (Leutzinger, 2022). In addition, recent work comparing multisensory, explicit approaches with more conventional methods reports stronger gains in reading and spelling for students with dyslexia when visual, auditory, and kinesthetic–tactile modalities are deliberately integrated into instruction (Saleem and Arshad, 2025). By drawing on these findings, the present study situates its remedial spelling program within the current evidence base on effective interventions for learners with documented spelling difficulties.

### Aims and objectives

The present study aimed to evaluate the effectiveness of a structured remedial spelling program for primary school students with LD. Specifically, it examined students’ spelling performance before and after participation in the program, investigated the extent to which spelling gains are retained over time, explores differences in improvement across subgroups with different types of LD, and identified the most effective instructional strategies for enhancing spelling skills in this population.

### Research questions

The study addresses the following research questions:

- Are there statistically significant differences between the control group’s pre- and post-test spelling scores?
- Are there statistically significant differences between the experimental group’s pre- and post-test spelling scores?
- Are there statistically significant differences between the experimental and control groups on the post-test spelling scores?
- Are there statistically significant differences between the experimental group’s post-test and follow-up spelling scores?

### Research significance

This research provides empirical evidence supporting the use of structured remedial spelling programs to improve literacy outcomes for students with LD. The findings offer practical guidance for teachers and special educators in refining instructional strategies, help curriculum developers embed systematic spelling support, and provide policymakers with a rationale for integrating targeted remedial interventions into mainstream education to promote equity and sustained academic success.

### Research hypotheses

Hypotheses were based on theoretical and empirical sources:

*H1*: There are no statistically significant differences between the control group’s pre- and post-test spelling scores.

*H2*: The experimental group’s post-test spelling scores are significantly higher than their pre-test scores.

*H3*: The experimental group scores significantly higher than the control group on the post-test spelling measure.

*H4*: There are no statistically significant differences between the experimental group’s post-test and follow-up spelling scores.

## Materials & methods

### Research design

The research employed a quasi-experimental design with a pre-test/post-test control group design (Maciejewski, 2020). This design allowed to compare spelling skills between an experimental group that received the remedial educational program and a control group that received no intervention. The chosen approach balanced practicality, feasibility, and ethical considerations. It allowed for assessing the intervention’s effectiveness while minimizing disruption to participants’ learning environments and ensuring equitable treatment. Despite the absence of random assignment, it maximizes internal validity by controlling for confounding variables and establishing causal relationships between the intervention and outcomes.

### Participants

Participants were 24 primary school students identified with LD in reading and/or writing, aged 9–12 years and enrolled in Grades 4–6, all of whom were officially placed in school-based learning-disability support programs following standard Ministry of Education eligibility procedures and were receiving specialized services at the time of the study. The presence of LD was confirmed through a combination of standardized psychoeducational assessment, review of school records, and teacher reports documenting persistent literacy difficulties despite appropriate classroom instruction; assessments included measures of intellectual functioning, reading and writing achievement, and neuropsychological skills relevant to literacy (for example, phonological processing, working memory, and visual–perceptual abilities), and indicated that most students showed marked weaknesses in phonological decoding, spelling accuracy, and reading fluency, with some also demonstrating pronounced difficulties in written expression. To ensure group comparability, students who met the inclusion criteria were first screened for equivalence in age, general cognitive ability, and socioeconomic background using an age-appropriate intelligence test and indices of family economic, social, and cultural status, after which the 24 eligible students were randomly assigned to an experimental group (*n* = 12) and a control group (*n* = 12); baseline analyses revealed no statistically significant differences between the two groups in age, IQ, neuropsychological performance, or economic, social, and cultural levels, indicating adequate initial equivalence prior to the intervention. The language of instruction in the participating classrooms was Arabic.

### Inclusion criteria

Participants eligible for inclusion in the study were primary school students diagnosed with LD that affected their performance in reading and/or writing. By focusing on students with documented LD in literacy, the study aimed to assess the effectiveness of the remedial education program in addressing spelling difficulties commonly associated with these LD.

### Exclusion criteria

Students with other significant disabilities that might have confounded the assessment of spelling skills were excluded from the study. This ensured that the findings applied specifically to the target population of students with LD in reading and writing, thereby enhancing the internal validity of the research.

### Instrumentation

The LD Spelling Scale LDSS was designed by the author as a study-specific instrument because available standardized spelling measures in the local context did not simultaneously target visual and phonetic spelling skills in primary-age students with LD in a way that matched the focus of the present intervention. These measures focus on broad dyslexia screening or general reading outcomes rather than providing a single, detailed measure that captures both visual–orthographic and phonological aspects of spelling in primary-age students (Arab, 2025; Gharaibeh, 2021). Its development was guided by commonly reported dimensions of spelling difficulty in this population, including weaknesses in phoneme–grapheme correspondence, visual discrimination of letter forms, application of basic orthographic patterns, and word analysis as highlighted in recent reviews of spelling instruction for students with or at risk for LD (Galuschka et al., 2020; Chandler et al., 2025). In line with recommendations for constructing progress-monitoring tools in special education, the LDSS items were written to reflect observable, classroom-relevant behaviors that teachers can rate consistently over time, with the primary aim of supporting research and growth tracking within this study rather than functioning as a fully standardized diagnostic test (Murnan, 2023; Speld QLD, 2025).

This measure developed for this study to evaluate spelling proficiency among primary school students with LD. It comprises 32 statements grouped into two dimensions; visual spelling and phonetic spelling, each containing 16 items that capture key aspects of phonics, vocabulary, spelling rules, and word analysis. Teachers rate students on a three-point scale (always, sometimes, never), generating systematic indicators of spelling performance across the two domains. The LDSS was administered at three time points (pre-test, post-test, and follow-up) to monitor change over the course of the intervention. Content validity was established through expert review: a panel of specialists examined the items for relevance, clarity, linguistic accuracy, and alignment with the construct of spelling skills in students with LD. Based on their feedback, items were refined through additions, deletions, and wording modifications, resulting in a final version that reflected expert consensus on the adequacy of the scale. Below are sample items from the LDSS that illustrate the phonetic and visual spelling skills assessed:

“The student pronounces alphabet sounds correctly. (Phonetic)”

“He differentiates letters with similar sounds. (Phonetic)”

“He writes letters with similar sounds correctly. (Phonetic)”

“He identifies the letter sound within the word. (Phonetic)”

“He matches the shape of the letter with its sound. (Visual)”

“He recognizes the shape of the letters within a word. (Visual)”

“He analyzes the word into its constituent letters. (Visual)”

“He writes the letters of the word in the correct order. (Visual)”

These sample phrases integrate common phonetic patterns, vocabulary words, and spelling rules suitable for primary school students, providing a comprehensive assessment of their spelling abilities. Each item on the scale is designed to evaluate spelling accuracy and proficiency within the context of meaningful sentences.

### The Statistical and Psychometric Proficiency of the Scale

The Statistical and Psychometric proficiency verification sample was 129 students with LD.

### Internal consistency

Internal consistency of the LDSS was examined using Pearson correlation coefficients at both the item and subscale levels. All items showed positive, statistically significant correlations with their respective subscale totals, and both subscales correlated strongly with the overall scale score (*p* < 0.01). These results, presented in Tables 1, 2, indicate satisfactory internal consistency for the LDSS.

**Table 1:** Correlation coefficients between the degree of each item and the total degree of dimension on the scale LDSS.

Phonetic spelling				Visual spelling			
N	Correlation coefficient	N	Correlation coefficient	N	Correlation coefficient	N	Correlation coefficient
1	0.678^**^	9	0.742^**^	17	0.619^**^	25	0.794^**^
2	0.744^**^	10	0.720^**^	18	0.560^**^	26	0.508^**^
3	0.585^**^	11	0.430^**^	19	0.622^**^	27	0.641^**^
4	0.773^**^	12	0.723^**^	20	0.634^**^	28	0.816^**^
5	0.560^**^	13	0.553^**^	21	0.643^**^	29	0.719^**^
6	0.764^**^	14	0.622^**^	22	0.591^**^	30	0.483^**^
7	0.756^**^	15	0.574^**^	23	0.428^**^	31	0.691^**^
8	0.776^**^	16	0.754^**^	24	0.499^**^	32	0.663^**^

All items have positive correlation coefficients and are statistically significant at the level of (0.01). ** Indicates statistical significance at *p* < 0.01.

**Table 2:** Correlations matrix of spelling scale.

N	Dimensions	First	Second	Total
1	Visual spelling	—		
2	Phonetic spelling	0.897^**^	—	
Total degree			0.967^**^	—

Significant at (0.01). ** Indicates statistical significance at *p* < 0.01.

It is clear from Tables 1, 2 that all correlation coefficients are significant at (0.01), which indicates that the LDSS has internal consistency.

### Reliability

#### Confirmatory factor analysis

**Figure 1:**
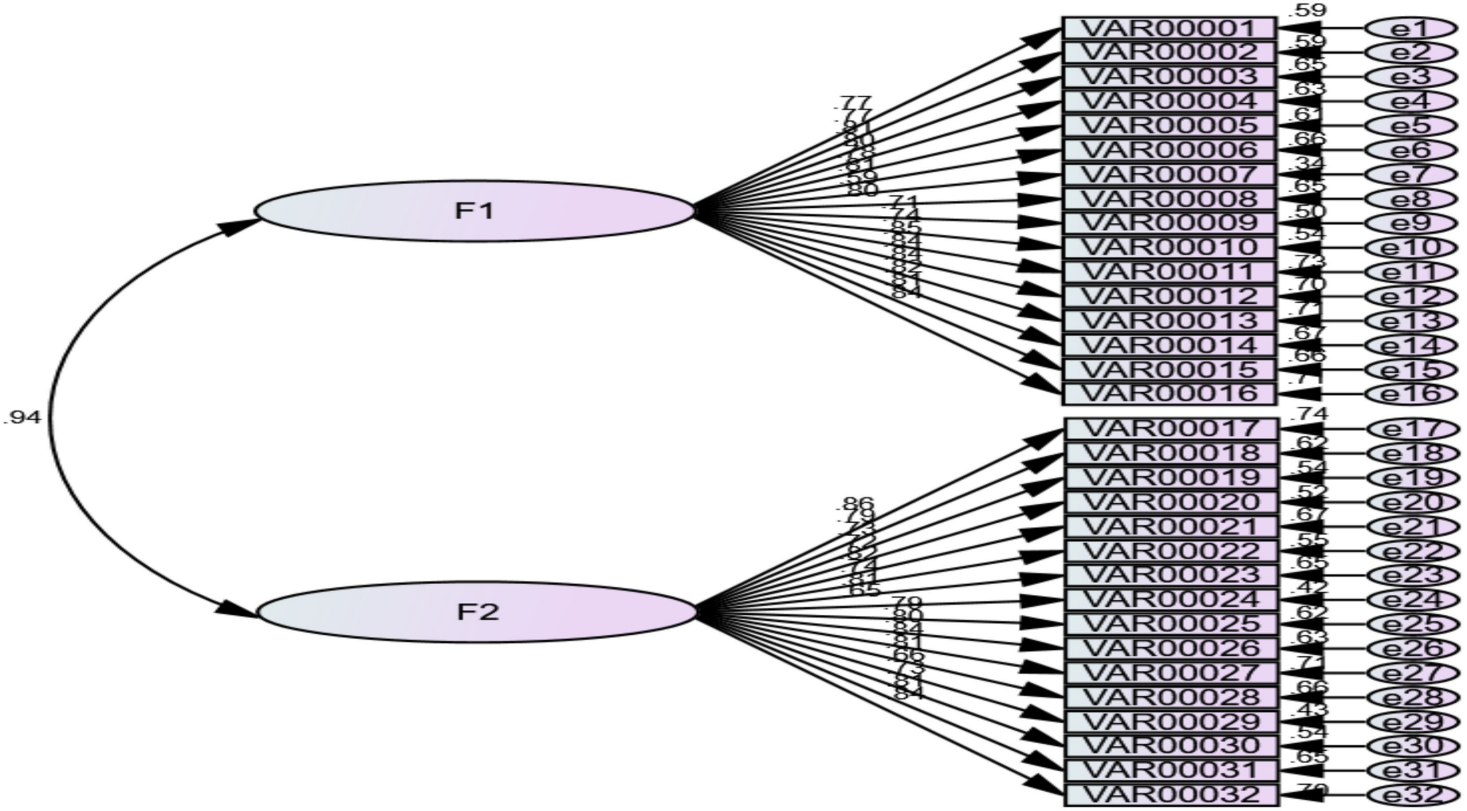
Model of the latent factors of the LDSS.The confirmatory factor analysis of the LDSS latent factors indicates good fit indices: χ² = 1620.800, df = 463, and RMSEA = 0.074, suggesting that the model demonstrates satisfactory construct validity.

The factor structure of the LDSS was analyzed in AMOS 26 to assess latent-level reliability and construct validity. A two-factor model was tested, with items loading on two correlated latent variables (visual spelling and phonetic spelling), as shown in Figure 1.

It has good identical indicators, where the value of (chi-square = 1620.800), freedom degree = 463, and the RMSEA index = 0.074, so the model has good identical indicators and Table 3 shows the results of the confirmatory factor analysis of the dimensions of the LDSS.

**Table 3:** Results of the confirmatory factor analysis.

Phonetic spelling				Visual spelling			
Observed factors	Saturation with latent factor	Standard errors of estimate	*T* values	Observed factors	Saturation with latent factor	Standard errors of estimate	*T* values
1	0.77	0.60	7.593^**^	17	0.86	0.74	7.215^**^
2	0.77	0.59	7.486^**^	18	0.79	0.62	7.574^**^
3	0.81	0.65	7.348^**^	19	0.73	0.54	7.686^**^
4	0.80	0.63	7.432^**^	20	0.72	0.52	7.704^**^
5	0.78	0.61	7.491^**^	21	0.82	0.67	7.417^**^
6	0.81	0.66	7.332^**^	22	0.74	0.55	7.669^**^
7	0.59	0.35	7.841^**^	23	0.81	0.65	7.493^**^
8	0.80	0.65	7.351^**^	24	0.65	0.42	7.807^**^
9	0.71	0.50	7.661^**^	25	0.79	0.62	7.572^**^
10	0.74	0.54	7.612^**^	26	0.80	0.63	7.539^**^
11	0.86	0.73	7.029^**^	27	0.84	0.71	7.297^**^
12	0.84	0.70	7.266^**^	28	0.81	0.66	7.479^**^
13	0.84	0.71	7.192^**^	29	0.66	0.43	7.779^**^
14	0.82	0.67	7.221^**^	30	0.74	0.54	7.694^**^
15	0.81	0.66	7.370^**^	31	0.81	0.65	7.473^**^
16	0.84	0.71	7.152^**^	32	0.84	0.71	7.330^**^

^**^Significant at 0.01.

Table 3 shows that the two-factor model achieved acceptable fit indices and that all factor loadings were significant at the 0.01 level, supporting the reliability of the LDSS. The findings indicate that the scale consists of two correlated latent factors-visual spelling and phonetic spelling-each represented by 16 observed items.

### Reliability

#### Test–retest method

This was done by re-applying the scale after 2 weeks on the same sample. Correlation coefficients were extracted using the Pearson coefficient (see Table 4).

**Table 4:** Reliability results with the re-test method.

Dimension	Correlation coefficients	Level of significance
Visual spelling	0.899	0.01
Phonetic spelling	0.728	0.01
Total degree	0.827	0.01

All coefficients are statistically significant at the 0.01 level, indicating that the scale produces stable scores over repeated administrations under similar conditions.

#### Cronbach’s alpha coefficient method

The reliability of the LDSS was estimated using Cronbach’s alpha, and the coefficients for both subscales and the total score were within the acceptable–good range (see Table 5).

**Table 5:** Reliability coefficients of the LDSS by using Cronbach’s alpha coefficient.

Dimension	Cronbach’s alpha coefficient
Visual spelling	0.767
Phonetic spelling	0.759
Total degree	0.797

Values indicate satisfactory internal consistency for the LDSS.

#### Split-half reliability

The LDSS was applied and corrected then divided into two halves, the first half included the odd-numbered phrases, and the second included the even-numbered phrases, for each student separately (see Table 6).

**Table 6:** Reliability coefficients of the LDSS using split-half method.

Dimension	Spearman–Brown coefficient	Guttman coefficient
Visual spelling	0.884	0.838
Phonetic spelling	0.851	0.812
Total degree	0.880	0.834

The Spearman–Brown and Guttman split-half coefficients for the LDSS were high and very similar across the scale dimensions, indicating strong internal consistency and supporting the reliability of the scale in measuring spelling performance among students with learning disabilities.

Based on the results obtained from the LDSS development and validation, which identified visual and phonetic spelling difficulties as key areas of weakness among students with LD, the remedial spelling program was designed to directly target these dimensions.

### The remedial educational program

The remedial program was designed to strengthen spelling performance in primary school students with LD affecting reading and/or writing. It comprised 18 sessions delivered three times per week over a six-week period, with each session lasting about 60 min. The instructional sequence moved systematically from foundational letter–sound relationships to more advanced word-level spelling skills, as outlined in Table 7. In each session, a clearly defined reading or spelling skill was targeted (such as letter–sound decoding, visual word recognition, word segmentation and blending, recognition of consonants and stressed letters, or writing words from memory), and the order of these skills was planned to support the stepwise development of accurate and fluent spelling.

**Table 7:** Sessions of the remedial educational program.

Sessions	Content	Aims
Session 1	Introduction to the remedial program	Welcome and introduction to the program Explanation of program objectives and expectations Motivating and encouraging students to learn Overview of spelling skills targeted
Session 2	Skill of letter-sound pronunciation	Recognize the exits of letters’ sounds Pronounce the letter sound correctly Distinguish between the letter sound and its shape
Session 3	Skill of writing letters from memory	Linking the letter shape with its name Master writing the alphabet from memory Differentiating similar pronunciation alphabet letters
Session 4	Skill of identifying a letter from a group of letters correctly	Knowing the name and shape of every letter Distinguish and differentiate between similar letters Reading every letter correctly
Session 5	Skill of recognizing the alphabet letters in their different forms	Know the alphabet letters in all their different positions Distinguish between different shapes of letters Read a group of words that contain alphabet letters in their different forms
Session 6	Skill of correct visual recognition of the word letters	Mention the word letters and explain the shape of the letter in all its positions Form a word from a group of letters and list the letters that make up the words
Sessions (7–8)	Skill of distinguishing pronunciation between similar letter sounds	Explain letter sounds that are similar in pronunciation Differentiate between similar letters Write words that consist of similar letters efficiently
Session 9	Skill of pronouncing the letter individually and then within a word	Pronounce the letter individually Recognize the shape of the letter and its different positions in the word Master the formation of meaningful words from letters
Sessions (10–11)	Skill of analyzing words into their main components	Explain symbols that make up the letters of a word Read and analyze words into their main components Feel the importance of recognizing the shape of the letter and the sound of the letter and linking them
Session 12	Skill of composing a word from a group of letters	Explain the alphabet letters in their various positions Reconstruct meaningful words from a group of letters Master writing words from memory
Session 13	Skill of pronouncing words orally	Be proficient in reading words orally Read words without pictures in front of them Feel the importance of recognizing words without aid
Session 14	Consonant recognition skills	Master the pronunciation of consonants Appreciate importance of pronouncing consonant within a word Write words containing consonants in different positions
Sessions 15–16	Skill of recognizing the stressed letter	Explain how to pronounce the stressed letter Read words that contain stress letters Explain the location of the stress when pronouncing
Session 17	Skill of imagining drawing the word after looking at it	Redraw the word from memory after looking at it Master writing words from memory Practice and master skill of visual-motor coordination
Session 18	Review and evaluation	Review of progress and achievements Evaluation of program effectiveness Discussion on next steps and continued support

The progression from basic letter–sound skills to more advanced word-level spelling and reading–writing integration skills.

Prior to implementation, the full program was reviewed by a panel of specialists to check the coherence between objectives, content, and training activities, as well as the linguistic accuracy of all materials, and their recommendations for revision were incorporated. Within the program, sessions alternated between reading and writing components so that gains in decoding and word recognition were closely linked to written production, minimizing any delay between the two strands. Each session followed a stable four-phase structure: introduction, presentation, evaluation, and closing. The introduction phase used a short, relevant question or prompt to focus attention and clarify the purpose of the target skill. During the presentation phase, the teacher delivered carefully structured activities aligned with pre-specified learning goals, using explicit modelling, scaffolding, and differentiation to respond to individual differences, while tracking each student’s progress, providing ongoing positive feedback, and using visual and technological supports to sustain engagement. The evaluation phase involved practice tasks and exercises directly tied to the skill taught in that session, followed by similar items to check understanding and consolidation. In the closing phase, key learning points were revisited, students’ efforts and successes were acknowledged, and their interest in participating in subsequent sessions was actively reinforced.

### Content of the sessions

The content, and aims of the remedial educational program are presented in Table 7.

### Data collection

The primary data collection method involves administering the (LDSS) as a pre-test, post-test, and follow-up assessment to measure spelling skills among primary school students with LD.

### Procedure

The study procedure consisted of four main steps, summarized in Table 8.

**Table 8:** The comprised procedure stages.

Step	Procedure	Group(s) involved	Purpose
1	Pre-test assessment	Experimental & Control	All participants completed the LDSS pre-test to establish baseline spelling skills
2	Intervention	Experimental	The experimental group received the remedial educational program; the control group did not
3	Post-test assessment	Experimental & Control	immediately after intervention Both groups completed the LDSS post-test immediately after the intervention period to measure spelling improvement
4	Follow-up assessment (2 months later)	Experimental	The experimental group completed the LDSS follow up 2 months later to evaluate retention of spelling skills

This structure provides a clear overview of the assessment and intervention timeline for both the experimental and control groups.

### Ethical considerations

Ethical approval for the study was obtained from the appropriate institutional review bodies prior to data collection, ensuring that all procedures complied with recognized ethical standards. Informed consent was obtained from the parents of all participating students, and confidentiality was safeguarded by assigning unique identification codes and storing all data securely so that no individual participant could be identified.

### Data analysis

Inferential statistics are utilized to make inferences or predictions about a population based on sample data (Trafimow and MacDonald, 2017). This involves analyzing the differences between groups to determine the impact of the remedial educational program. The statistical program (AMOS 26) and the SPSS statistical packages were used, including the Mann–Whitney test, Wilcox on signed ranks test, matched-pairs rank biserial correlation, and Cronbach’s alpha to compare the experimental and control groups in the pre-test, post-test and follow-up assessment. These statistical tests determine whether the two groups significantly differ in spelling skills before and after the intervention.

This analysis helps evaluate the effectiveness of the remedial educational program by examining changes in spelling skills within each group over time. ANOVA is utilized to explore differences in spelling scores among subgroups based on specific variables. These statistical tests assess whether there are significant differences in spelling skills across different subgroups within the sample population (Breitsohl, 2019).

### Baseline equivalence between the experimental and control groups

The sample comprised 24 students with LD, aged from nine to twelve years old, with a mean age 10.75, and a standard deviation was 1.03. Baseline equivalence between the experimental and control groups was examined on background variables, including age, IQ, neuropsychological assessment scores, and economic, social, and cultural levels, as shown in Table 9.

**Table 9:** Baseline equivalence between the experimental and control groups.

Variables		Group	Mean	Std. deviation	Mean rank	Sum of ranks	*U*	*Z*	Sig.
Age		Exp.	10.67	1.15	12.00	144.00	66.0	0.368	n.s.
		Con.	10.83	0.94	13.00	156.00			
IQ Factor		Exp.	101.42	3.73	12.13	145.50	67.5	0.262	n.s.
		Con.	101.67	3.47	12.88	154.50			
Neuropsychological assessment		Exp.	64.58	1.00	12.21	146.50	68.5	0.213	n.s.
		Con.	64.75	1.06	12.79	153.50			
Economic, social and cultural level	Economic level	Exp.	39.33	1.07	11.79	141.50	63.5	0.507	n.s.
		Con.	39.58	1.24	13.21	158.50			
	Social level	Exp.	15.25	0.87	12.33	148.00	70.0	0.124	n.s.
		Con.	15.33	0.98	12.67	152.00			
	Cultural level	Exp.	7.67	0.65	11.92	143.00	65.0	0.438	n.s.
		Con.	7.83	0.83	13.08	157.00			
	Total	Exp.	62.25	1.71	11.83	142.00	64.0	0.472	n.s.
		Con.	62.75	2.18	13.17	158.00			

Exp. refers to the experimental group; Con. refers to the control group.

There were no statistically significant differences between the experimental and control groups on any of the baseline background measures (age, IQ, neuropsychological assessment, and economic, social, and cultural levels), indicating that the two groups were equivalent prior to the intervention (see Table 10).

**Table 10:** Baseline equivalence between the experimental and control groups in spelling.

Dimensions	Group	Mean	Std. deviation	Mean rank	Sum of ranks	*U*	*Z*	Sig.
Visual spelling	Exp.	18.25	0.97	11.50	138.00	60.0	0.729	n.s.
	Con.	18.58	1.00	13.50	162.00			
Phonetic spelling	Exp.	18.17	0.72	10.92	131.00	53.0	1.203	n.s.
	Con.	18.50	0.67	14.08	169.00			
Total degree	Exp.	36.42	0.90	10.25	123.00	45.0	1.752	n.s.
	Con.	37.08	1.16	14.75	177.00			

There were no statistically significant differences between the groups in spelling skills.

## Results

### Differences between the control group’s pre- and post-test spelling scores

To examine whether the control group showed any change in spelling performance over time, the Wilcoxon “W” test was used to compare pre-and post-test scores on the LDSS dimensions (visual spelling, phonetic spelling, and total score) (see Table 11).

**Table 11:** The pre- and post-tests for the control group on the LDSS.

Dimensions	Assessment	Mean	Std. deviation	Ranks	*N*	Mean rank	Sum of ranks	*p*	Sig.
Visual spelling	Pre-test	18.58	1.00	− + =	3 6 3	6.00 4.50	18.00 27.00	0.543	n.s.
	Post-test	18.83	1.03						
Phonetic spelling	Pre-test	18.50	0.67	− + =	1 5 6	3.00 3.60	3.00 18.00	1.667	n.s.
	Post-test	18.92	0.67						
Total degree	Pre-test	37.08	1.16	− + =	2 5 5	3.50 4.20	7.00 21.00	1.207	n.s.
	Post-test	37.75	1.06						

There were no statistically significant differences indicating that spelling performance remained stable over time which confirmed the validity of the first hypothesis.

### Differences between the experimental group’s pre-and post-test spelling scores

To evaluate change in the experimental group’s spelling performance, the Wilcoxon “W” test was conducted comparing pre- and post-test LDSS scores (see Table 12).

**Table 12:** The pre-and post-tests of the experimental group on the LDSS.

Dimension	Assessment	Mean	Std. deviation	Ranks	*N*	Mean rank	Sum of ranks	*Z*	Sig.	N_2_
Visual spelling	Pre-test	18.25	0.97	− + =	0 12 0	0.00 6.50	0.00 78.00	3.088	0.01	0.891
	Post-test	39.50	1.83							
Phonetic spelling	Pre-test	18.17	0.72	− + =	0 12 0	0.00 6.50	0.00 78.00	3.074	0.01	0.887
	Post-test	39.33	2.53							
Total degree	Pre-test	36.42	0.90	− + =	0 12 0	0.00 6.50	0.00 78.00	3.066	0.01	0.885
	Post-test	78.83	3.35							

The results indicated statistically significant improvements in visual spelling, phonetic spelling, and total spelling scores at post-test compared with pre-test (all *p* < 0.01), demonstrating substantial gains in spelling performance following the remedial program which supported the second hypothesis.

### Differences between experimental and control groups on post-test spelling scores

**Figure 2:**
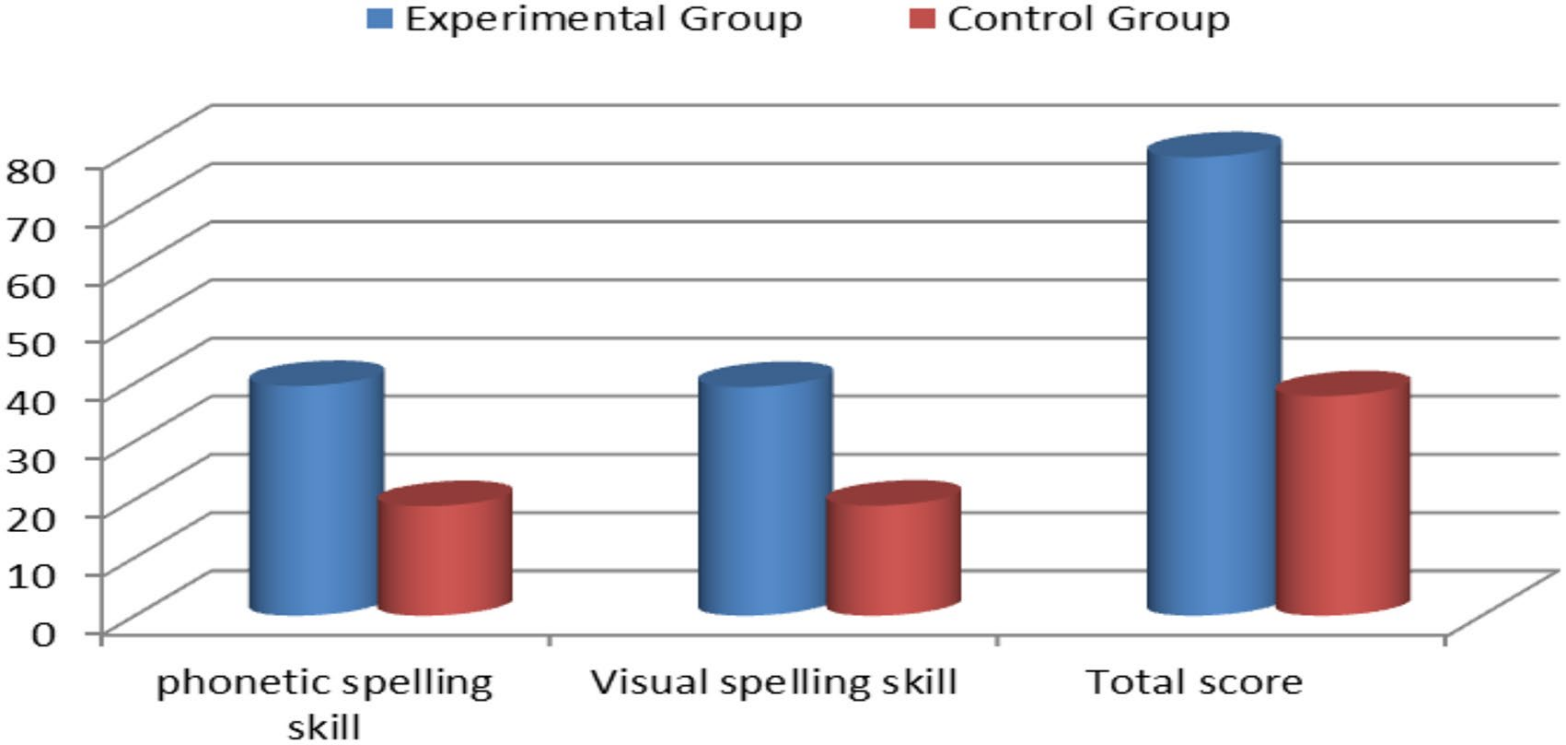
Differences between the experimental and control groups in the post-test.

Between group differences at post-test were examined using the Mann–Whitney *U* test on LDSS visual spelling, phonetic spelling, and total scores (see Table 13 and Figure 2).

**Table 13:** The post-test for the experimental and control groups.

Dimensions	Assessment	Mean	Std. deviation	Mean rank	Sum of ranks	*Z*	Sig.
Visual spelling	Exp.	39.50	1.83	18.50	222.00	4.193	0.01
	Con.	18.83	1.03	6.50	78.00		
Phonetic spelling	Exp.	39.33	2.53	18.50	222.00	4.219	0.01
	Con.	18.92	0.67	6.50	78.00		
Total degree	Exp.	78.83	3.35	18.50	222.00	4.190	0.01
	Con.	37.75	1.06	6.50	78.00		

The experimental group obtained significantly higher post-test scores than the control group on all LDSS dimensions, indicating that students who received the remedial program outperformed those who did not.

### Differences between the experimental group’s post-test and follow up spelling scores

To assess the maintenance of spelling gains in the experimental group, the Wilcoxon “W” test was used to compare LDSS post-test and follow up scores on visual spelling, phonetic spelling, and the total scale (see Table 14).

**Table 14:** The post and follow-up for the experimental group.

Dimensions	Assessment	Mean	Std. deviation	Ranks	*N*	Mean rank	Sum of ranks	*Z*	Sig.
Visual spelling	Post-test	39.50	1.83	− + =	5 5 2	4.70 6.30	23.50 31.50	0.412	n.s.
	Follow up	39.75	1.71						
Phonetic spelling	Post-test	39.33	2.53	− + =	5 5 2	4.90 6.10	24.50 30.50	0.308	n.s.
	Follow up	39.50	2.50						
Total degree	Post-test	78.83	3.35	− + =	5 6 1	5.90 6.08	29.50 36.50	0.313	n.s.
	Follow up	79.25	3.25						

There were no statistically significant differences between post-test and follow up scores on LDSS dimension (all *p* > 0.05), suggesting that the improvements achieved through the remedial program were maintained over the 2 months follow up period which confirmed the validity of the fourth hypothesis.

## Discussion

The discussion section interprets the findings of the present study in light of existing research on LD, spelling assessment, and remedial instruction, and highlights their implications for practice and future research. In what follows, the discussion is organized into three parts: first, the development and validation of the LDSS as a tool for assessing visual and phonetic spelling skills are considered; second, the outcomes of the remedial spelling program are examined; and third, a general discussion integrates insights from both components to outline their combined contribution to supporting students with LD in spelling.

### The development of LDSS

The LDSS provided a structured and sensitive measure of visual and phonetic spelling skills in primary-age students with LD, capturing the types of orthographic and phonological weaknesses typically reported in this population (Fletcher et al., 2018; Graham, 2019). The distinction between visual–orthographic features (for example, recognizing and sequencing letter forms) and phonological features (for example, mapping sounds to letters and discriminating similar phonemes) is consistent with evidence that spelling difficulties in LD often arise from combined deficits in orthographic and phonological processing (Stanovich and Siegel, 1994; Wanzek et al., 2006). Baseline LDSS scores in the present sample indicated substantial initial impairment on both dimensions, suggesting that the scale is sensitive to the profile of students with literacy-related LD and can inform the design of targeted interventions (Miciak and Fletcher, 2020).

These findings align with calls for assessment tools that go beyond global spelling scores to provide multidimensional profiles that can guide instruction (Graham and Santangelo, 2014; Truckenmiller et al., 2014). In this study, LDSS scores functioned both as outcome measures and as a diagnostic framework for understanding the specific nature of students’ spelling difficulties, which strengthens the interpretation of subsequent intervention effects (Graham et al., 2017; Seidenberg, 2013). At the same time, because the LDSS was developed and validated with a relatively context-specific sample, further research is warranted to replicate its factor structure, reliability, and validity with larger and more diverse populations and to examine convergence with other standardized spelling and word-level literacy measures (Santangelo and Graham, 2016; Wanzek et al., 2018).

### The effectiveness of the remedial intervention

The intervention findings provide clear support for the effectiveness of the remedial educational program in improving spelling skills among students with LD. Baseline analyses showed that the experimental and control groups did not differ significantly on age, IQ, neuropsychological measures, or socioeconomic indicators, which offers a strong basis for attributing subsequent group differences in spelling performance to the intervention rather than to pre-existing disparities (Fletcher et al., 2018; Miciak and Fletcher, 2020). The experimental group demonstrated significant pre- to post-test gains on LDSS visual and phonetic spelling scores, while the control group showed no meaningful change, and post-test comparisons favored the experimental group on all spelling dimensions. This pattern aligns with evidence that explicit, systematic spelling instruction can substantially improve spelling accuracy and related literacy outcomes for students with LD (Graham and Santangelo, 2014; Santangelo and Graham, 2016; Wanzek et al., 2006).

The demographic profile and baseline equivalence of the sample further support the internal validity of the study. Participants were primary-stage students with formally identified LD and comparable cognitive and socioeconomic characteristics, which reflects samples commonly described in research on literacy interventions for this population (Fletcher et al., 2018; Goodwin and Ahn, 2013). The absence of significant pretest differences and the lack of improvement in the control group suggest that the robust gains observed in the experimental group are unlikely to be attributable to maturation or test practice effects and instead reflect the impact of the remedial program (Graham et al., 2017; McArthur et al., 2012).

Several design features of the program appear to account for its effectiveness. Instruction was explicit, cumulative, and carefully sequenced, with each session targeting clearly specified spelling goals and building on prior learning—an approach repeatedly shown to benefit students with LD (Archer and Hughes, 2011; Gillespie Rouse, 2024). The program was individualized, with instructional tasks and supports adjusted to the needs of each student, which is consistent with research indicating that interventions for students with LD are most effective when they combine direct instruction with strategic, student-responsive adaptations (Gersten et al., 2009; Swanson and Hoskyn, 2001).

Behavioral and organizational supports were also central components. The use of positive reinforcement and clear verbal guidance helped strengthen desired spelling behaviors, while breaking tasks into manageable steps, supported by verbal and visual cues, reduced cognitive load and increased the likelihood of successful engagement (McLeskey et al., 2017; Gillespie Rouse, 2024). The deliberate use of simple, precise language further facilitated comprehension and is consistent with recommendations that students with learning difficulties benefit from scaffolded support that includes written, spoken, and visual prompts (Capp, 2017; CAST, 2018). The contrast between the substantial gains in the experimental group and the minimal change in the control group underscores the added value of such intentionally designed, scaffolded instruction over generic classroom practices (Graham, 2019; Nathalie and Kathy, 2019).

The multisensory nature of the program is another likely mechanism of change. Activities that engaged visual, auditory, and tactile modalities—such as manipulating magnetic or textured letters and saying while writing—helped students internalize letter–sound relationships and common spelling patterns, in line with evidence that multisensory, explicit approaches produce stronger reading and spelling outcomes for students with LD (Ehri et al., 2017; Saleem and Arshad, 2025; Santangelo and Graham, 2016). The incorporation of assistive technologies, including text-to-speech, word prediction, and speech-to-text tools, provided ongoing scaffolding for practice, self-checking, and error correction, reinforcing targeted skills and likely contributing to the higher LDSS post-test scores in the experimental group (Alqahtani, 2020; Swanson et al., 2015).

The positive psychological climate established during the sessions appears to have amplified these instructional components. Early sessions focused on building rapport, explaining the goals and relevance of the program, and creating a supportive environment, which encouraged regular attendance and active engagement—conditions that have been linked to better outcomes in literacy interventions for students with LD (Graham et al., 2017; Santangelo and Graham, 2016). The maintenance of spelling gains at follow-up suggests that the program produced durable changes in spelling competence rather than short-lived practice effects, echoing findings that well-designed, sustained interventions are necessary to achieve lasting benefits for this population (Nathalie and Kathy, 2019; Wanzek et al., 2018).

### Synthesis of LDSS and program outcomes

Taken together, the LDSS development and the remedial intervention offer an integrated assessment–intervention framework for supporting students with LD in spelling. The LDSS provided a psychometrically supported means of profiling visual and phonetic spelling difficulties and monitoring change over time, while the remedial program translated this diagnostic information into structured, multisensory, and behaviorally informed instruction that produced substantial and sustained gains in spelling (Graham and Santangelo, 2014; Santangelo and Graham, 2016). These two components are mutually reinforcing: precise assessment allows teachers to identify specific deficits in orthographic and phonological processing, and targeted instruction then addresses those deficits directly, creating a coherent pathway from diagnosis to intervention and evaluation (Fletcher et al., 2018; Wanzek et al., 2018).

At a broader level, the findings contribute to ongoing discussions about how to operationalize inclusive education for students with LD. By using an instrument such as the LDSS to generate detailed learner profiles and then designing a remedial program that is explicit, cumulative, and multisensory, the study exemplifies an individualized, needs-based approach that is increasingly recommended in policy and practice documents on inclusive schooling (Serry et al., 2022; Gillespie Rouse, 2024). The documented gains in spelling, coupled with the maintenance of these gains at follow-up, suggest that when assessment and intervention are tightly aligned, students with LD can achieve meaningful and durable improvements in foundational literacy skills, with likely downstream benefits for participation, self-efficacy, and broader academic engagement (Fletcher et al., 2018; Wanzek et al., 2018). In this sense, the present study illustrates how reliable assessment combined with carefully designed intervention can help mainstream schools move toward more equitable outcomes for students with LD in spelling, and it highlights the importance of embedding such assessment–intervention systems within routine practice rather than treating them as add-on or short-term projects.

### Implications

The findings of this study have several important implications for educational practice and future research. First, the marked improvement in spelling skills among the experimental group underscores the value of tailored, evidence-based interventions for students with LD, and suggests that similar programs could be adopted more widely in school settings. Second, the pattern of sustained gains over time indicates the importance of continuous monitoring and flexible adjustment of instructional strategies, so that support can be refined in response to individual students’ progress (McMaster et al., 2014). Third, the successful outcomes of the remedial program align with contemporary inclusive education principles, highlighting the role of supportive, adaptable learning environments that accommodate diverse learner profiles (Shapiro, 2018). In addition, the maintenance of benefits at follow-up points to the need for longitudinal research that examines the longer-term impact of such interventions on broader academic performance. Future studies should also investigate other factors that may shape spelling development, such as family involvement and classroom climate, in order to refine and target interventions more precisely. Finally, complementing quantitative outcomes with qualitative methods—such as interviews and classroom observations—could deepen understanding of students’ experiences and needs, and inform the design of more effective and empathetic remedial programs (Sullivan and Artiles, 2011).

## Conclusion

The present research investigated the effectiveness of a remedial educational program in enhancing spelling skills among primary school students with LD. Through statistics, the study provided valuable insights into the participants’ demographic characteristics, the intervention’s efficacy, and the differences in outcomes between the control and experimental groups.

The findings revealed that the program significantly improved spelling skills among students with LD. Participants in the experimental group demonstrated notable enhancements in post-test scores compared to the control group, highlighting the effectiveness of targeted interventions. The analysis further confirmed the significant differences in scores between the control and experimental groups, underscoring the transformative potential of tailored interventions in promoting academic success among students with LD.

### Recommendations

Based on the significant and sustained improvement in spelling skills observed among students who participated in the remedial program, it is recommended that evidence-based remedial programs of this type be incorporated into school policies and practice to provide systematic support for students with LD in spelling. The findings also point to the importance of preparing teachers to recognize, differentiate, and respond to spelling-related learning difficulties, so that similar targeted interventions can be implemented and monitored effectively in regular classrooms. In addition, the pattern of gains and their maintenance over time suggests a need for further research that builds directly on this program, examining how specific components of the intervention work for different learner profiles and how these components can be refined to address particular challenges in spelling.

### Future implications

- Explore the potential of emerging technologies for personalized and engaging interventions, considering the positive impact observed with technology-based tools.
- Support longitudinal studies to assess the sustained impact of remedial programs on spelling proficiency and broader academic success.
- Promote inclusive practices that address the unique needs of students with diverse LD, fostering a more supportive and adaptive educational environment.

### Limitations

Despite the promising findings, several limitations should be acknowledged. Firstly, the study’s sample size was relatively small, which may limit the generalizability of the findings to broader populations of students with LD. Secondly, the analyses involved multiple statistical comparisons, which may increase the risk of Type I error, even though the number of primary outcomes was restricted and a consistent analytical strategy was used. In addition, the study focused solely on spelling skills, overlooking potential improvements in other areas of academic performance, and the short-term nature of the follow-up assessment may not capture the long-term sustainability of the intervention’s effects. Future research should address these limitations by employing larger samples, assessing a broader range of academic outcomes, using analytic approaches that better control for multiple testing, and conducting longer-term follow-up assessments.

## Data Availability

The raw data supporting the conclusions of this article will be made available by the authors, without undue reservation.
